# Robust dynamical pattern formation from a multifunctional minimal genetic circuit

**DOI:** 10.1186/1752-0509-4-48

**Published:** 2010-04-22

**Authors:** Guillermo Rodrigo, Javier Carrera, Santiago F Elena, Alfonso Jaramillo

**Affiliations:** 1 Instituto de Biología Molecular y Celular de Plantas, CSIC-UPV, 46022 Valencia, Spain; 2 Synth-Bio group. Epigenomics Project, Genopole-Université Évry Val d'Essonne-CNRS UPS3201, 91034 Évry Cedex, France; 3 Instituto ITACA, Universidad Politécnica de Valencia, 46022 Valencia, Spain; 4 Santa Fe Institute, Santa Fe, NM 87501, USA; 5 Ecole Polytechnique, Laboratoire de Biochimie, 91128 Palaiseau Cedex, France

## Abstract

**Background:**

A practical problem during the analysis of natural networks is their complexity, thus the use of synthetic circuits would allow to unveil the natural mechanisms of operation. Autocatalytic gene regulatory networks play an important role in shaping the development of multicellular organisms, whereas oscillatory circuits are used to control gene expression under variable environments such as the light-dark cycle.

**Results:**

We propose a new mechanism to generate developmental patterns and oscillations using a minimal number of genes. For this, we design a synthetic gene circuit with an antagonistic self-regulation to study the spatio-temporal control of protein expression. Here, we show that our minimal system can behave as a biological clock or memory, and it exhibites an inherent robustness due to a quorum sensing mechanism. We analyze this property by accounting for molecular noise in an heterogeneous population. We also show how the period of the oscillations is tunable by environmental signals, and we study the bifurcations of the system by constructing different phase diagrams.

**Conclusions:**

As this minimal circuit is based on a single transcriptional unit, it provides a new mechanism based on post-translational interactions to generate targeted spatio-temporal behavior.

## Background

Synthetic Biology aims to engineer genetic networks with defined dynamics [1]. For this, it usually relies on the use of design principles derived from the analysis of natural genetic networks. Those networks are large and complex systems with many unknown interactions that can dramatically affect the system dynamics. Then, for a complete understanding of the mechanisms underlying gene networks it is valuable the engineering of synthetic circuits that have a minimal complexity. In addition, such small circuits would allow the modular design of complex hierarchical structures with targeted spatial and temporal behaviors. However, even the design of small circuits with existing genetic components is very challenging due to the lack of enough parameters to fine-tune the system. In fact, the use of properly characterized genetic components favors an accurate prediction of the dynamics of an *in vivo *implemented circuit [2-5]. The extreme case being the design of a genetic network composed of a single transcriptional unit showing a specified spatio-temporal dynamics. As all the protein concentrations shall be coupled, it is very difficult to have a non-trivial dynamics unless the time scales of protein interactions and of cell-to-cell communication are conveniently coupled.

In higher organisms, development results from the coordinated action of thousands of genes at any moment during the cell cycle. However, small regulatory circuits control the execution of genetic programs by triggering cell differentiation according to spatial patterns [6]. These patterns result from gradients of signaling molecules, which diffuse in the medium and are sensed at each moment by the cell circuitry. Quantitative models based on reaction-diffusion equations have been successfully applied to understand the principles of organism's development [7-9]. Furthermore, synthetic patterns have been previously engineered in bacteria [10] and flies [11]. However, genetic systems with defined spatial and temporal behavior have not been artificially constructed yet. In such a synthetic system, the fate of every cell within the population could be controlled, for instance, by oscillators working in a specific manner in response to spatial location or by the state of an internal memory. It is of particular interest to apply the same design principles underlying naturally occurring molecular clocks, where rythmicity is mainly based on negative feedback loops [12], to the *in vivo *engineering of synthetic oscillatory circuits [13,14].

**Figure 1:**
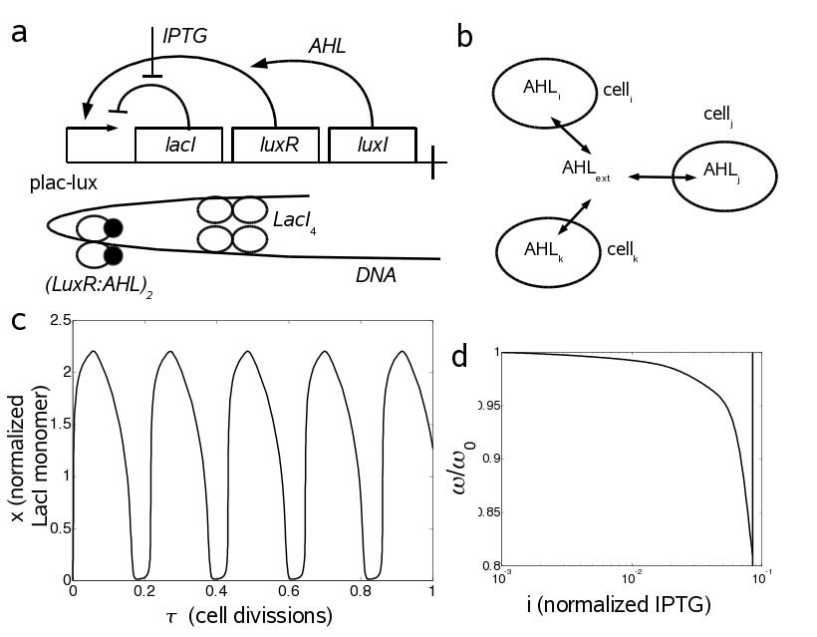
**Scheme of the system and dynamical simulation at the single cell level**. (a) Scheme of the synthetic gene cassette and the fully regulated promoter forming a delay-inducing DNA loop. Arrows (blunt lines) mean positive (negative) regulations. (b) Quorum sensing scheme. Each cell produces AHL which is pumped to the medium, allowing cell population to reach a homogeneous concentration of AHL. (c) Simulation of the circuit behavior at the single cell level showing oscillations with the nominal parameters (Table 1). The initial condition is given by no molecular species in the system and with no IPTG in the medium. (d) Normalized oscillatory frequency versus the amount of IPTG (vertical line gives the limit where the sustained oscillations are lost, *i*_*c *_≃ 0.085).

The simplest imaginable genetic circuit consists in a single operon with a feedback loop. On the one hand, negative autoregulation promotes robustness [15], but it can also cause oscillations if the process introduces a delay [16-18]. On the other hand, positive autoregulation yields bistability [19]. By combining both structures, we have designed and analyzed theoretically a synthetic genetic circuit with a minimal transcription structure exhibiting multifunctionality (Fig. 1a). We present a mathematical model at the molecular level based on differential equations for the synthetic self-regulated transcription circuit. The system shows oscillatory and bistable behaviors, together with intrinsic robustness through a quorum sensing (QS) mechanism (Fig. 1b) that allows for cellular synchronization [20,21]. The system, which is expressed from plasmids, consists of two transcription factors (TFs) responding to two different chemicals. Thus, we perform spatio-temporal simulations showing different dynamic pattern formation depending on the initial environment.

## Results and Discussion

The system, a single transcriptional unit, consists in a combinatorial promoter, lactose-luciferase, which controls the expression of two TFs LacI and LuxR, and the enzyme LuxI (see Methods for further details). Being all the concentrations of protein species proportional, it would make *a priori *especially difficult our targeted dynamics. Fortunately, we can still have a rich dynamics at single cell owed to the suitable design of molecular interactions (multimerization and binding events). Furthermore, this model is coupled to a population model, where cell-to-cell communication introduces the spatial dimension. Usually the models including the spatial dimension require the use of several genes with uncoupled dynamics. Here we will show that a dynamical pattern behavior can be generated by using genes expressing the same concentration of proteins (up to a proportionality factor).

### Multifunctional behavior

Using experimentally measured parameter values (Table 1), we simulate the dynamic behavior of the system (Fig. 1c). The period of the oscillations is about 20 min, which is 5-fold shorter than the cell doubling period (*τ*_0 _= 100 min). This period can be environmentally tuned without genetic modifications of the circuit. We show the evolution of the frequency of oscillations (*ω*), normalized by the nominal one from Fig. 1c (*ω*_0_), for different concentrations of IPTG (Fig. 1d), resulting in a losing of oscillations at high levels of IPTG (*i *>*i*_*c*_) due to the inactivation of the negative loop. In addition, higher values of the enzymatic degradation coefficient (*δ*) yield higher *ω *because of the rapidity of the transients (data not shown).

**Table 1:** Kinetic parameters used in the spatiotemporal transcription model.

Parameter	Ref.	Parameter	Ref.
*α *= 125 nM/min	[35]	*θ*_*Y *_= 10 nM	[10]
Λ = 75 nM	[26]	*θ*_*X *_= 5 nM	[22]
*δ *= 300 nM/min	[26]	*θ*_*I *_= 15 *μ*M	[23]
*L*_0 _= 4.1	[23]	*T *= 5.5 min	[22]
*K*_1 _= 100 nM	[2]	*K*_2 _= 10 nM	[2]
*K*_3 _= 55 nM	*	*K*_4 _= 300 nM	*
*κ *= 10 nM/min	[32]	*η *= 2000 min^-1^	[33]
*δ*_*A *_= 0.002 min^-1^	[30]	*λ*_*A *_= 1 min^-1^	[30]
*N*_*m *_= 10^5 ^cells/nL	*	*D *= 10^-3 ^mm^2^/min	[10]

In this work, we consider *C *= 50, *σ *= 1, *τ*_0 _= 100 min^-1^, *φ *= 100, and Ω = 1. *This value was estimated from the literature.

**Figure 2:**
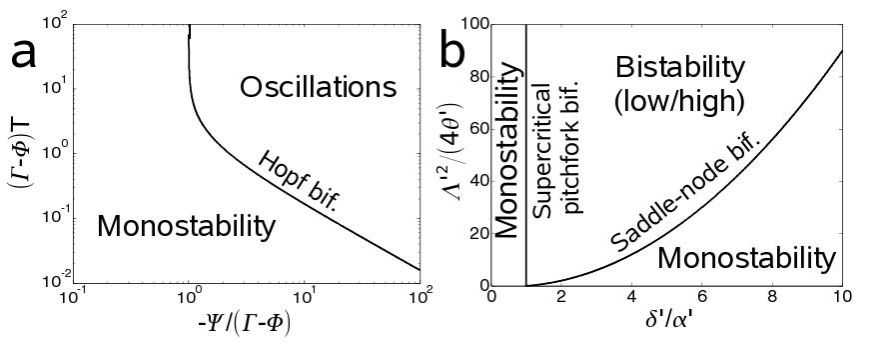
**Stability analysis of the dynamic genetic circuit at the single cell level**. (a) Oscillatory region of the system at very low levels of IPTG (*i *= 0). Φ, Ψ, and Γ are defined in the main text. The bifurcation for bistability is at -Ψ/(Γ - Φ) = -1 (not shown in the Figure). (b) Bistability region of the system at very high levels of IPTG (*i *>> 1) and external AHL (*a *>> 1). To plot (b) we have considered a simplified model (see Methods).

**Figure 3:**
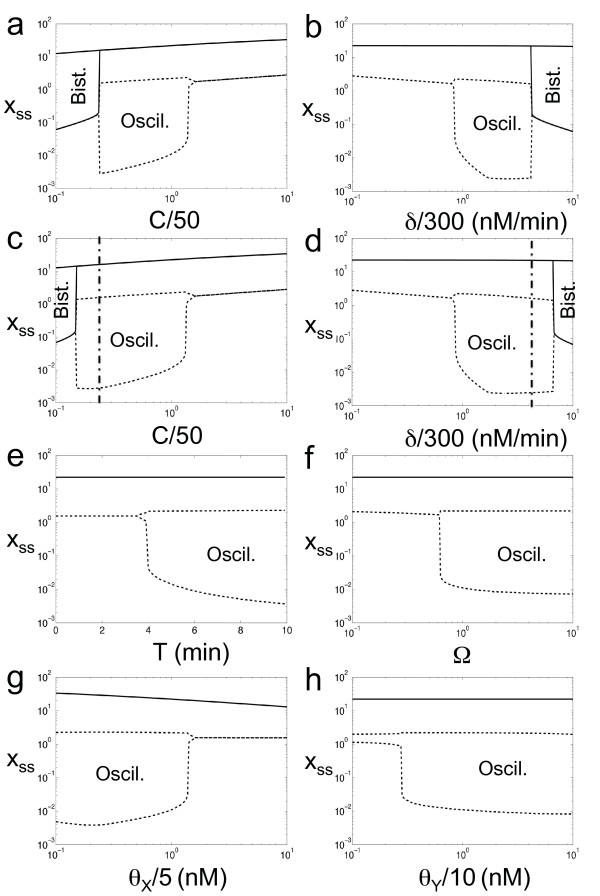
**Sensitivity analysis of the circuit for the most outstanding parameters**. The rest of the parameter values are shown in Table 1. We plot the steady state(s) (*x*_*ss*_) at very high levels of IPTG, *i *= 10^3 ^(solid lines), and with no IPTG (dashed lines). Bifurcation of solid lines indicates the bistability limit condition. Bifurcation of dashed lines represents the oscillatory limit point, where we plot the maximal and minimal values of the oscillatory dynamics. In c, d AHL is externally introduced at very high levels.

We analyze the functional sensitivity of the system. In Fig. 2a we plot the phase diagram showing the Hopf bifurcation. The delay and the nonlinearity of the repressive loop enhance the oscillatory behavior. Contrarily, at high concentration of IPTG the system is stable and it can reach bistability according to the bifurcation diagram shown in Fig. 2b. Furthermore, we study the sensitivity of the dynamical behavior to different kinetic parameters of the model (Fig. 3). We have selected the most relevant ones to study the dynamical properties of steady states reached. We show a robust oscillatory region, which would give strong chances for a successful mode of action. Certainly, the ranges for the appropriate kinetic parameter values are sufficiently large to ensure a potential biological implementation.

**Figure 4:**
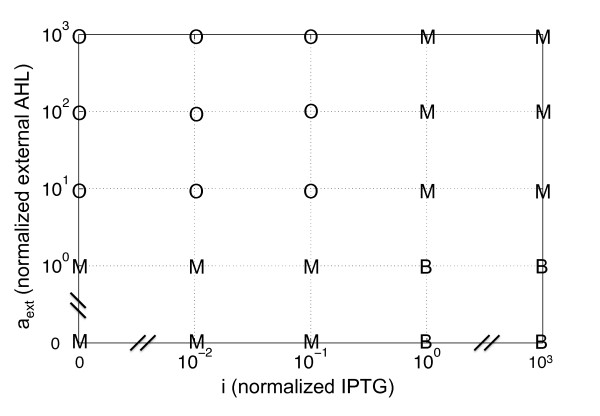
**Stability analysis of the circuit as a function of IPTG and external AHL (assumed constant)**. Herein, we consider a low plasmid copy number (*C *= 10). We plot the dynamical regimes of the circuit (M, monostability; B, bistability; O, oscillations) for different values of the external inducers.

Firstly, we have carried out an analysis of the effect of the transcription and degradation terms. These terms are proportional to the plasmid copy number (*C*) and the enzymatic degradation coefficient (*δ*), respectively. On the one hand, in Fig. 3a we can observe oscillatory and bistability regions. High values of *C *yields monostability. However, intermediate values yields oscillations at low levels of IPTG, whereas low values yields bistability at high levels of IPTG. Importantly, the external addition of high amounts of AHL enhances the oscillatory region in detriment to bistability (Fig. 3c). This fact allows a transition between oscillations and bistability using IPTG and AHL. To better illustrate this transition, we have performed a stability analysis of the circuit, with *C *= 10, using IPTG and external AHL as control parameters (Fig. 4). The map shows four different regions: bistable at high levels of IPTG and low of external AHL, oscillatory at low IPTG and high external AHL, and monostable in the other two cases. On the other hand, the circuit is monostable for low values of *δ*, oscillatory for intermediate values and low levels of IPTG, and bistable for high values and high levels of IPTG (Fig. 3b). Analogously to the previous case, the external addition of AHL enhances the oscillatory region for high values of *δ*, thus allowing a transition between the oscillatory and bistability regimes (Fig. 3d). Additionally, in Fig. 3e, we show how the delay (*T*) is necessary to reach the oscillatory regime. In that sense, the minimal required delay is about 4 minutes (for the nominal values of the kinetic parameters shown in Table 1). In this work we have considered a delay of 5.5 minutes, based on the estimation of the unlooping kinetic constant in 0.18 min^-1 ^[22]. According to Fig. 3f, cooperative binding between LacI_4 _and (LuxR:AHL)_2 _(Ω > 1) enhances the oscillatory regime, whereas competitive binding (Ω < 1) makes the system stable. In the natural case of LacI_4 _and CRP_2_:cAMP, the value of Ω was estimated in 10.3 [23]. Moreover, in Figs. 3g, h we analyze the protein-DNA binding coefficients for the two TFs of the circuit (*θ*_*X *_for LacI_4 _and *θ*_*Y *_for (LuxR:AHL)_2_). They have antagonistic effects for the dynamical behavior of the circuit, that is, lower values of *θ*_*X *_and higher of *θ*_*Y *_enhance oscillations. This fact increases the experimental chances to implement such a circuit, since LacI is considered a strong repressor and the activation capacity of LuxR will not be decisive for the proper function of the circuit.

Interestingly, our circuit does not need further genetic manipulation to change the behavior regime. Environmental signals control the dynamics leading a fully tunable circuit, since by varying the concentration of IPTG and AHL we can change the dynamical regime. According to our model, this minimal genetic unit can display complex dynamics. The integration in a single circuit of the ability of oscillating and having memory may have important applications in Synthetic Biology.

### Synchronization of oscillations

**Figure 5:**
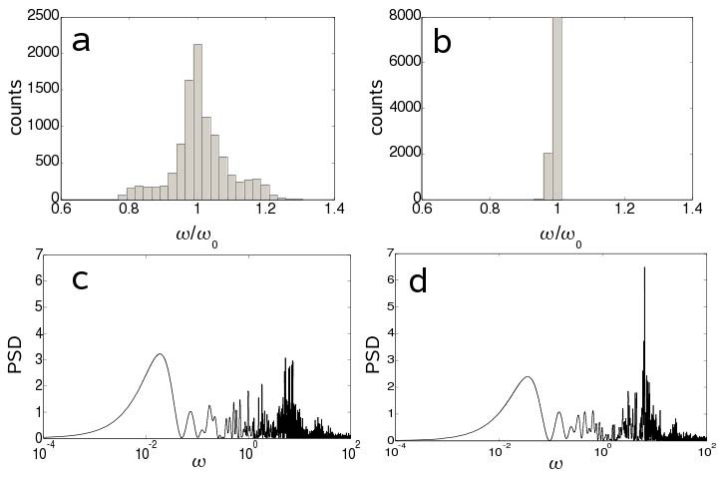
**Frequency histogram of the system response (a) without and (b) with quorum sensing**. To plot those histograms, *C *and *δ *are assumed to be Gaussian random numbers with mean 50 and 300 respectively and standard deviation 10. Power spectral density (PSD) analysis by considering a white noise (c) without and (d) with quorum sensing. Figures are obtained at very low levels of IPTG (*i *= 0).

As we have a population of biological clocks coupled through a diffusive molecule, it is very interesting to analyze the onset of synchronization of such oscillators. We address this at local level, without considering spatial features. The equilibrium between the extra and intracellular species is much faster than their diffusion in the medium, thus it is reasonable to uncouple these two transport mechanisms to study the synchronization by QS. Moreover, we have performed such simulations with no IPTG in the medium. Firstly, we have considered a heterogeneous population where each cell has a different number of plasmidic copies (*C*) and enzymatic degradation coefficient (*δ*). Thus, these parameters are assumed Gaussian random numbers, *C *= *N *(50, 10) and *δ *= *N*(300, 10). Poisson distributions give equal results (data not shown). Following the frequency histograms shown in Figs. 5a, b, QS provides a population synchronization as the variance of the distribution with QS compared to the case without QS is significantly smaller. This fact suggests that genetic mutations that eventually affect the kinetic properties of the circuit and occur in all single individuals within the population, but in an uncorrelated way, are compensated in terms of population. Importantly, synchronization by QS relies on symmetrical distributions around a nominal value. However, QS will fail to synchronize when mutations always affect the model parameters in the same sense.

Secondly, we have introduced an intrinsic white noise to account for the stochasticity raised from the small number of molecules. It is important to notice that this noise is always present in a discrete system of molecules, although at high number of molecules it is usually neglected. We have not accounted for external sources of noise. Power spectral density (PSD) analysis has been performed showing that QS enhances regular oscillations, since in that case there is one frequency with a PSD significantly higher (Figs. 5c, d). The global effect of AHL together with the different temporal scales in the system, first producing the activator then the repressor with a delay, enables to sustain in time the oscillatory behavior of the whole population.

### Spatio-temporal patterns

**Figure 6:**
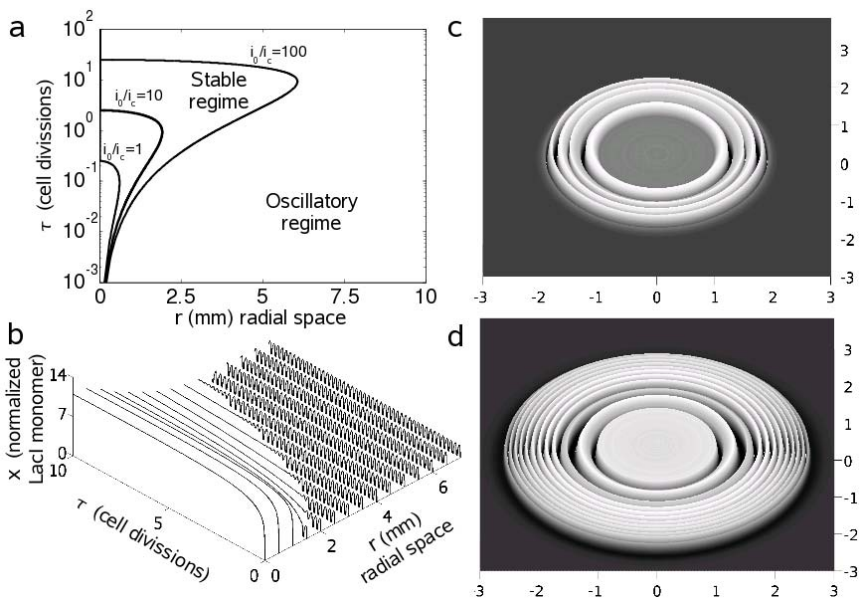
**Dynamical pattern formation**. (a) Spatio-temporal diagram showing the different regimes of operation (front given by *r*_*c*_). (b) Dynamical behavior in response to an external signal of IPTG (*I*(*r *= 0, *τ *= 0) = 10^6 ^nM). (c and d) Spatial plot at different times (*τ *= 1 and *τ *= 3) showing the generated patterns when AHL is externally introduced but not produced (*κ *= 0) nor degraded (*λ*_*A *_= 0) into the cells (*A*(*r *= 0, *τ *= 0) = 10^4 ^nM). We use a white/black scale denoting high/low concentrations.

The fact of having an oscillating circuit allows the obtaining of patterns with dynamical behavior. The possibility of producing waves in genetic systems opens the door to the development of new types of tissue engineering that could adapt to the environment. On the one hand, In Figs. 6a, b we show the spatio-temporal pattern evolution triggered by IPTG. An initial stable state of high LacI is reached quickly and it propagates to neighboring cells outwards in radial direction as IPTG diffuses. At large radius we have low IPTG that gives oscillations until the concentration of IPTG reaches the critical level (*i*_*c*_). This composed structure can be viewed as a spatial pulse that filters oscillations into constant signals. On the other hand, in Figs. 6c, d AHL is externally introduced at high levels to generate patterns that change with time as AHL diffuses. The spatial structure of concentric rings dynamically changes by varying the protein expression level at each point and adding more rings. At low radius, there is a monomodal transition from low to high expression levels, indicating that the amount of AHL is above certain threshold that controls gene expression in that region. By assuming a non-homogeneous diffusion, we could generate non-symmetrical structures. Each cell carries an oscillator whose period depends on the spatial location and time.

The *in vivo *implementation of such a circuit and its characterization could be the matter of further work, beyond the scope of this study. Experimentally, a fluorescent protein such as GFP could be inserted into the operon as reporter. Time-lapse microscopy could be used to visualize fluorescence in solid-phase in a scale of various cm in space and tens of generations in time [10], which would be sufficient to observe the dynamical patterns. Even though, this could also be tested readily with the use of microfluidic devices. In that way, the experimental work would help to validate or refine the mathematical model.

## Conclusions

In this manuscript, we have shown for the first time the design of a single trancriptional unit with a post-translational dynamics that couples spatial and temporal scales to generate dynamical spatial structures. We have followed a model-based design approach to obtain a minimal genetic unit with multiple functionalities and displaying certain homeostasis to both environmental and mutational perturbations, since external fluctuations or variations in kinetic parameters are compensated due to QS. We have relied on nonlinear dynamics and stochastic modeling to analyze our system under biological noise. We have provided a new mechanism able to switch from oscillatory to bistable regimes using an external inducer and to produce complex spatio-temporal patterns by using a single transcriptional unit. In addition, this sort of small functional modules could be hierarchically assembled to generate more complex systems [24]. That these simple units are able to generate such a complex behavior provides new avenues to understand natural genetic circuits by designing synthetic minimal systems. The bottleneck in the construction of a synthetic network consists in the number of independent transcriptional units, as transcription carries the largest source of intrinsic and extrinsic noises [25]. Although a system consisting in a single operon would be very appealing, it may have serious problems due to its minimalism unless the system is properly designed. In the past, most authors have used at least two operons to construct systems with oscillatory behavior or producing a patterning. Although for the former, it was already known several examples of oscillatory circuits consisting in a single operon. That our minimal circuit can display such a rich behavior highlights the fact that rational design techniques take advantage of engineering principles for constructing genetic circuits with specified functions.

## Methods

### Dynamics at the single cell level

A synthetic lactose-luciferase (lac-lux) promoter controls the transcription of the lac repressor LacI (*X*), the activator LuxR (*Y*) and the enzyme LuxI (*Z*). Here, we consider the bacterium *Escherichia coli *as cellular chassis, and all proteins are assumed to be ssrA-tagged for enzymatic degradation [26], which for a zeroth-order kinetics enhances robustness of oscillations [27]. In addition, the chemical IPTG (*I*) acts as inducer and binds to LacI inhibiting its repressive effect; the chemical AHL (*A*) is required by LuxR to activate transcription. The active form of LacI is a tetramer (*X*_4_), whereas for LuxR it consists of a dimmer of the complex LuxR-AHL ((*Y:A*)_2_). In addition, repression by LacI tetramer induces a DNA loop in the promoter region (1a) [28], which may introduce a delay (*T*) into the system. The processes of transcription, translation, folding and multimerization could also induce a delay, but this is neglected in this work. Indeed, transcription in eukaryotes dictates a delay because of splicing [16]. However, this is not the case in prokaryotes. Herein, we assume that the reaction between two tetramers for making the DNA loop is not reversible, given that the looping structure remains even for low levels of LacI [22].

Furthermore, here we assume a fast mRNA dynamics (as the quasi-steady state is roughly reached in 5 min) [29]. The total concentrations of the three proteins (*X*, *Y *and *Z*) remain proportional, then we reduce the model to a single variable. The dynamics of the regulatory model is given by(1)

where *C *is the plasmid copy number, *α *the nominal transcription/translation rate, *δ *and Λ the kinetic constants of the ClpXP protease, *μ *the cell growth rate, and *σ *the ratio activator/repressor (*X *= *σY *= *σZ*, being (1 + 2*σ*)*X *the total protein amount). LacI-mediated DNA loop enhances the autorepression. This is incorporated into the model by introducing an additional repressive term proportional to a looping constant (*L*_0_). In case of no loop, *L*_0 _= 0. Moreover, this repression can be modulated by (LuxR:AHL)_2_, as it occurs with CRP_2_:cAMP in the natural lac promoter [23]. The functional form of the regulatory factor *f *has been previously studied [23,28] and is given by(2)

where *θ*_*X *_is the binding coefficient of LacI_4_-DNA, *θ*_*I *_of LacI-IPTG_2_, *θ*_*Y *_of (LuxR:AHL)_2_-DNA, and 1/*φ *accounts for the basal transcription rate. The parameter Ω accounts for the potential interaction of LacI_4 _and (LuxR:AHL)_2 _in the DNA loop. In case of no interaction, Ω = 1. In addition, the synthesis rate of AHL is assumed proportional to LuxI, and we consider that cells express the enzyme AiiA which degradates AHL [30](3)

where *κ *is the synthesis constant of AHL by LuxI, *δ*_*A *_the thermodynamic degradation constant of AHL, and *λ*_*A *_the degradation rate by AiiA.

The multimerization kinetics (at a given total cellular amount of AHL) is given by(4)

where *k*_*l*/-*l *_are the forward/reverse kinetic coefficients (*l *= 1, 2, 3, 4). By exploiting the different time scales in the dynamics and neglecting the amount of DNA-bound protein, we can define the dimensionless variables as *x *= *X*_1_/*θ *with , *i *= *I*/*θ*_*I*_, *y *= *Y*_1_/(*K*_4_*θ*_*Y*_)^1/2^, and *a *= *A/K*_3_. Notice that *y *and *a *depend on *x*. The reactions for multimerization can be assumed much faster than the ones for transcription and translation. Then the system (4) is reduced to the steady state. Being that, we obtain , *Y:A *= *Y*_1_*A/K*_3_, and , with *K*_*l *_= *k*_-*l*_/*k*_*l*_.

Furthermore, the total amounts of LacI and LuxR are *X *= *X*_1 _+ 2*X*_2 _+ 4*X*_4 _and *Y *= *Y*_1 _+ *Y:A *+ 2(*Y:A*)_2 _respectively. For simplicity in the notation, we denote *X *as a function of the dimensionless variable *x*, , and . In addition, we assume that the total amount of AHL is approximately equal to the free one, then it turns out that . Being that, *Y:A *= 0 when *A *= 0 and  at very high levels of *A*. For the following, we denote *x*_*T *_= *x*(*t *-*T*). Time is also re-scaled by cell division, *τ *= *μ*_0_*t/ln*(2) = *t*/*τ*_0_.

Accordingly, the regulatory and degradation factors read, respectively, as(5)

### Spectral analysis

We define  and . Then, the differential equation (1) reads(6)

for a constant value of *i*. We denote ,  and , where these variables are evaluated in the steady state (*x*_*ss*_). The steady state satisfies . Being Δ*x *= *x *-*x*_*ss*_, using first-order perturbations we can write(7)

Thereby, the equation of eigenvalues (*λ*) is *λ *= Φ -Γ + Ψ*e*^*-λT *^[31]. Let us define the following variables  and *U*_2 _=(Γ - Φ)*T *to simplify the results of the spectral analysis. The oscillatory boundary condition is given by *λ *= *jω*, being *j *the imaginary unit. Then we obtain , the analytical equation for the Hopf bifurcation. On the contrary, the bistability boundary condition implies *λ *= 0, then we obtain *U*_1 _= -1. To further analyze the bistability of the system, we simplify the model at high levels of IPTG and externally introduced AHL. Without lost of generality, a dynamics governed by  captures the principal features of the system, where *α' *~*Cα*, *θ' *~*σ*^2 ^*K*_4 _*θ*_*Y*_/*θ*^2^, *δ' *~*δ*, and Λ*' *~Λ.

### Numerical integration and stochasticity

To illustrate this point, let us consider a molecular system governed by the following general differential equation , where *X *is the protein amount. The transcription term is highly nonlinear and accounts for the system delay (*T*). In our case, the function *f *depends on both *X*(*t*) and *X*(*t *-*T*). For the enzymatic degradation, we have to notice that, as Λ is a low value, this becomes zeroth-order for high values of *X*, while first-order when *X *is close to 0.

The delay-based system can be numerically integrated following(8)

To solve this equation we use the MATLAB routine dde23.

To account for molecular noise we use the Langevin approach [25]. We introduce an intrinsic white noise into the model(9)

where *η *(*t*) is a random fluctuation with ⟨*η *(*t*)⟩ = 0 and ⟨*η *(*t*)*η *(*t'*)⟩ = *δ *(*t*- *t'*) (Dirac delta). This equation was solved using the specified routine and considering a constant noise in the integration interval (Δ *t*)(10)

where *ξ *is a Gaussian-distributed random number with mean 0 and standard deviation .

### Dynamics at the population level

LuxI catalyzes the production of AHL which can be pumped to the medium facilitating a cell-to-cell communication (i.e., QS) [32]. We also account for the dynamics of the intracellular and extracellular (labeled with subscript *e*) concentrations of IPTG and AHL, and for the cell population (*N*, with ) together with the spatial role in solid medium. The diffusion, the intracellular and the extracellular dynamics are given by(11)

where *η *the equilibrium constant of the membrane transport of IPTG and AHL [33], *v *= *V/V*_*e *_the ratio of volumes, *D *the diffusion (assumed linear and homogeneous) coefficient, and *N*_*m *_the maximum cell capacity of the medium. Being the transport through the cell membrane fast, it turns out that *I *≃ *I*_*e *_and *A*_*e *_≃ *QA *with(12)

Since AHL is quickly degraded and *Q *≃ 1 in a large population, we can take the quasi-steady state *A *= *κZ/λ *_*A *_when AHL is not externally introduced. In addition, we neglect the movement of cells when replicating because even for *τ *= 100 this displacement would be ~0.1 mm.

Then, integrating the molecular and population models, the reaction-diffusion dynamics of the dimensionless cellular system is governed by(13)

where the space is normalized by *D*. Solving the partial differential equation for IPTG diffusion in time and radial space (*r*) [34], the signaling pattern reads(14)

where *r*_0 _is the radius of the initial drop of IPTG (here assumed 1 mm) and *I*_0 _is the modified Bessel function. Since for a given concentration of IPTG (*i*_*c*_) the system is not oscillatory, we obtain, using the equation (14), the spatio-temporal limit(15)

where, for small values of *r*_0 _(*r*_0 _<<*Dt*), we have assumed that .

## Authors' contributions

GR and AJ conceived this study and designed the circuit. GR developed the mathematical model and carried out the simulations. JC participated in the model construction and performed some simulations. AJ and SFE supervised the work. All authors participated in writing the manuscript. All authors read and approved the final manuscript.
